# The Landscape of Tumor Dissemination in Leptomeningeal Disease: Mapping CTC Plasticity and Regional Heterogeneity Across Blood and CSF Compartments

**DOI:** 10.3390/cancers18193124

**Published:** 2026-09-26

**Authors:** Stephanie N. Shishido, Baishali Chaudhuri, Vidur Mushran, Kena Ihle, Yujun Wang, Colt A. Egelston, James Hicks, Lisa Feldman, Eliza Barragan, Peter Kuhn

**Affiliations:** 1 Michelson Center for Convergent Bioscience, Convergent Science Institute in Cancer (CSI-Cancer), University of Southern California, Los Angeles, CA 90089, USA; sshishid@usc.edu (S.N.S.);; 2 City of Hope National Medical Center, Duarte, CA 91010, USA; 3 USC Norris Comprehensive Cancer Center, Keck School of Medicine, University of Southern California, Los Angeles, CA 90033, USA; 4 Department of Biological Sciences, Dornsife College of Letters, Arts and Sciences, University of Southern California, Los Angeles, CA 90089, USA; 5 Department of Aerospace and Mechanical Engineering, Viterbi School of Engineering, University of Southern California, Los Angeles, CA 90089, USA; 6 Department of Biomedical Engineering, Viterbi School of Engineering, University of Southern California, Los Angeles, CA 90089, USA; 7 Catherine and Joseph Aresty Department of Urology, Institute of Urology, Keck School of Medicine, University of Southern California, Los Angeles, CA 90033, USA

**Keywords:** leptomeningeal disease, breast cancer, circulating tumor cells, liquid biopsy, cerebrospinal fluid, single-cell genomics, copy number variation, warm autopsy, regional heterogeneity, cellular plasticity

## Abstract

**Background/Objectives:** Leptomeningeal disease (LMD) is among the most severe complications of metastatic breast cancer, with median survival measured in weeks. Definitive diagnosis relies on neuroimaging and cerebrospinal fluid (CSF) cytology, which carries a false-negative rate of 40–50% at a single lumbar puncture. Improved tools for detecting and characterizing tumor cells across systemic and central nervous system (CNS) compartments are urgently needed. **Methods:** We applied an enrichment-free, fluorescence whole-slide imaging (fWSI)-based liquid biopsy platform to ante-mortem and post-mortem peripheral blood (PB) and CSF from five patients with metastatic breast cancer and confirmed or suspected LMD, collected through the City of Hope LEGACY warm autopsy program. Circulating tumor cells (CTCs) were classified by immunophenotype (cytokeratin [CK], vimentin [VIM], CD45/CD31) and morphology. Select cells underwent single-cell copy number variation (CNV) profiling by low-pass whole-genome sequencing. **Results:** Across 23 samples (11 PB, 12 CSF), we identified marked compartmental heterogeneity in CTC burden, phenotype, and genomic architecture. In the one patient (Patient 1) sampled across all four ventricular-to-cisternal CSF sites, CTC frequency declined from proximal to distal compartments (11.4% in the right lateral ventricle to 0.6% at the foramen magnum), a single-patient observation consistent with a proximal-to-distal gradient. Patients with active, untreated LMD showed high CSF CTC incidence with both epithelial and mesenchymal phenotypes, whereas the two patients receiving intrathecal therapy each showed low or absent CSF CTCs; given the small numbers involved and a plausible technical confound in a third patient, we interpret this association cautiously. Single-cell CNV profiling of CSF-derived CTCs across patients with active LMD revealed highly concordant chromosomal aberration profiles, 100% of CK+ CSF CTCs analyzed carried chromosomal aberrations, consistent with, though not formally establishing, expansion of a genomically evolved subclone within the leptomeningeal compartment; PB-derived cells showed substantially greater heterogeneity. In the two patients with paired post-mortem arterial and venous PB, arterial blood showed higher total rare cell counts in both, and higher CK-positive CTC incidence in one (1425.73 vs. undetectable epi.CTC/mL); the other showed the reverse for this specific metric. **Conclusions:** Enrichment-free, multi-compartment liquid biopsy reveals a regionally heterogeneous and biologically structured landscape in breast cancer LMD not resolvable by conventional cytology or single-site venous sampling. These findings are consistent with a hypothesis-generating model in which the leptomeningeal space acts as a selective niche for clonally convergent tumor expansion. They also point to the potential of regionally resolved CTC profiling for monitoring LMD treatment response in future, larger cohorts.

## Simple Summary

Leptomeningeal disease (LMD) is a devastating complication of metastatic breast cancer that is difficult to detect and monitor with current diagnostic tools. In this study, we used an imaging-based liquid biopsy approach to analyze tumor cells in blood and cerebrospinal fluid (CSF) from five patients with breast cancer and confirmed or suspected LMD, collected through the City of Hope LEGACY warm autopsy program. We found that tumor cell burden, appearance, and genetic makeup varied substantially across different fluid compartments sampled from the same patient. In the CSF, tumor cells showed highly similar genetic alterations consistent with clonal expansion within the leptomeningeal space, whereas blood contained more diverse tumor cell populations. Additionally, the two patients who received intrathecal (into-the-spinal-fluid) therapy in this small study had low or absent tumor cells in their CSF, suggesting this may be a promising avenue to monitor treatment, though this needs to be confirmed in more patients. These findings reveal that multi-compartment liquid biopsy can capture biologically meaningful differences in tumor cell distribution that single-site sampling would miss, offering new opportunities to improve diagnosis and monitoring in this difficult-to-treat disease.

## 1. Introduction

Leptomeningeal disease (LMD) represents one of the most devastating manifestations of metastatic cancer, arising when malignant cells disseminate to and propagate within the cerebrospinal fluid (CSF) and leptomeningeal surfaces of the central nervous system [1,2]. Despite advances in systemic therapy and neuroimaging, outcomes for patients with LMD remain poor, in part due to delayed diagnosis, limited tools for disease monitoring, and an incomplete understanding of how tumor cells traffic between systemic and central nervous system (CNS) compartments. LMD affects approximately 5% of patients with breast cancer [3,4] and occurs more frequently in patients with triple-negative and other high-risk breast cancer subtypes [1,4]. Current diagnosis relies primarily on neuroimaging and conventional CSF cytology in conjunction with clinical evaluation of symptoms [5]. While imaging can suggest leptomeningeal involvement, sensitivity is variable, and cytologic detection of malignant cells in CSF is notoriously unreliable, often necessitating repeated lumbar punctures, with a false-negative rate of 40–50% at a single lumbar puncture [6]. Current treatments combine targeted therapies (such as trastuzumab deruxtecan or tucatinib), intrathecal chemotherapy often delivered via an Ommaya reservoir, and radiation [4,5]. Despite these advances, outcomes remain poor. Improved liquid biopsy strategies capable of sensitively detecting and characterizing tumor-associated cells in both peripheral blood (PB) and CSF could substantially enhance diagnostic accuracy and provide new insights into disease biology.

Liquid biopsy approaches in LMD have historically focused on cell-free analytes, including circulating tumor DNA and RNA [7]. While informative, these analytes provide limited regional and phenotypic context. In contrast, circulating tumor cells (CTCs) and other rare cellular populations preserve morphological, protein, and molecular features that reflect tumor state, lineage plasticity, and interactions with the immune microenvironment [8,9]. However, most CTC technologies rely on enrichment strategies that may bias detection toward predefined phenotypes and are poorly suited for low-volume or low-cellularity samples such as CSF. To address these challenges, we used an enrichment-free, imaging-based liquid biopsy platform that enables comprehensive cataloging of all nucleated cells within a sample. This approach allows for unbiased detection and classification of rare cellular events, including epithelial and mesenchymal CTCs, immune cells, and other atypical populations, while preserving regional and morphological information.

Enrichment-based CTC platforms (e.g., EpCAM selection, size filtration) introduce phenotypic biases that may exclude mesenchymal or immune-mimicry CTC populations and are particularly ill-suited to the low-volume, low-cellularity CSF matrix [10]. This fWSI-based approach addresses these limitations: all nucleated cells are immobilized on glass slides without selection, stained by multiplex immunofluorescence, and scanned by high-speed whole-slide imaging, enabling unbiased cataloging of all rare cellular events with recorded regional coordinates for downstream single-cell genomic characterization [11,12,13,14].

Furthermore, the regional distribution of tumor cells across CSF compartments is not uniform. Tumor cells shed at the ventricular tumor-CSF interface are subject to progressive dilution, immune-mediated clearance, and mechanical attrition as they flow rostrally to caudally through the CSF, with intact CTCs particularly vulnerable compared to smaller cell-free analytes [15,16,17]. Clinical data demonstrate CTC or ctDNA detection rates approaching 100% in intraoperative CSF, approximately 93% in ventricular samples, and approximately 66% in lumbar-derived CSF [15]—a gradient that can reverse when LMD burden is concentrated in the spinal canal [16,17], consistent with reports in other CNS tumor types that liquid-biopsy detection sensitivity depends on tumor localization relative to the sampling site [18]. These findings underscore the need for regionally resolved, multi-compartment sampling strategies.

In this study, we leveraged samples collected through the City of Hope LEGACY warm autopsy program [19], a unique framework that enrolls patients with end-stage metastatic breast cancer while alive and collects biofluids ante-mortem during treatment. Within 6–8 h of death, the program enables large-volume post-mortem collection from multiple anatomically distinct blood and CSF compartments [19], allowing us to examine liquid biopsy cellularity across multiple anatomical compartments in patients with metastatic breast cancer and clinically suspected or confirmed LMD. By analyzing paired ante-mortem and post-mortem PB and CSF samples, including regionally distinct CSF compartments, we sought to (i) define compartment-specific differences in cellular composition, (ii) assess how these differences relate to clinical course and treatment exposure, and (iii) explore the potential biological and clinical significance of regional heterogeneity in LMD.

## 2. Materials and Methods

### 2.1. Study Design and Patient Cohort

This observational study was conducted as part of the LEGACY warm autopsy program, approved under City of Hope IRB protocols 17,503 and 20,649 [19]. Patients with advanced metastatic breast cancer and clinical suspicion or diagnosis of leptomeningeal disease were enrolled under an institutional review board–approved protocol with written informed consent. Peripheral blood and CSF samples were collected either ante-mortem, post-mortem, or both, depending on clinical circumstances and patient participation. Post-mortem collections enabled sampling from multiple anatomical compartments, including arterial and venous blood, as well as CSF obtained from distinct CNS locations such as the right and left lateral ventricles, fourth ventricle, Ommaya reservoir, and foramen magnum. Clinical data, including tumor subtype, sites of metastatic disease, treatment history (including intrathecal therapy), and cause of death, were abstracted from medical records.

### 2.2. Sample Collection and Processing

CSF samples were collected in EDTA tubes and processed within 24 h of collection as previously described [20]. CSF samples were obtained either through Ommaya reservoirs or direct ventricular and cisternal access at autopsy. Total cell counts were measured to guide downstream processing. PB samples were collected in Streck cfDNA tubes and processed within 48 h of collection. Red blood cells were removed by ammonium chloride–based lysis, and nucleated cells were recovered by centrifugation. Cells were resuspended in buffered solution and plated as a monolayer onto custom glass slides (Marienfeld Superior, Lauda-Königshofen, Germany, Cat. No. 0650000) at approximately 3 million cells per slide. For peripheral blood, multiple slides were generated per sample to accommodate higher cellularity, whereas all nucleated cells from CSF samples were plated onto a single slide. Slides were stored at −80 °C until staining and imaging.

### 2.3. Immunofluorescence Staining and fWSI

Slides were thawed and incubated at 37 °C for 40 min prior to fixation. Slides were then fixed, permeabilized, and blocked with bovine serum albumin (BSA) prior to immunofluorescence staining. A multiplex antibody panel was used to label epithelial markers (cytokeratins; CK), mesenchymal markers (vimentin; VIM), hematopoietic and endothelial markers (CD45/CD31; CD), and nuclear DNA (DAPI) [11]. Following staining, slides were scanned using automated high-speed fluorescence microscopy to generate multi-channel whole-slide images [11,12]. The marker-fluorophore-display color key used consistently across all figures is: CK, Alexa Fluor 555 (red); VIM, Alexa Fluor 488 (white); CD45/CD31, Alexa Fluor 647 (green); DAPI (blue).

### 2.4. Liquid Biopsy Cellular Detection and Classification

Two complementary computational approaches were used for cellular detection. For peripheral blood samples, a rare-cell detection pipeline identified candidate events based on morphometric and fluorescence outlier analysis relative to the background leukocyte population, as previously described [13,14,20].

For CSF samples, which exhibit lower background cellularity, all nucleated cells were digitally cataloged in two stages. In the first stage, nuclei and candidate CK-positive cells were independently segmented using Otsu and triangle thresholding, respectively, followed by watershed-based separation. Candidate nuclei and cell bodies were filtered on morphology (size, circularity, solidity) and fluorescence intensity thresholds. In the second stage, each candidate CK-positive cell was verified for nuclear co-localization within the cell body mask; candidates lacking a qualifying internal nucleus or showing artifactual DAPI signal overlap were discarded. Confirmed cells were mapped to their slide coordinates using a per-slide nuclear density deposition mask. The pipeline was implemented in Python version 3.10 using scikit-image version 0.22, SciPy version 1.11, and NumPy version 1.26.

For all samples, trained analysts subsequently reviewed candidates to confirm cellular integrity, morphology, and marker expression. Within the combined CD45/CD31 (‘CD’) fluorescence channel, CD45 and CD31 are not spectrally distinguishable; CD45+ leukocyte versus CD31+ endothelial/platelet identity was therefore resolved by trained analysts on the basis of morphology and staining pattern rather than the channel alone. CD45+ leukocytes present with a compact, cell-body-filling CD signal and the small, round, densely DAPI-stained nucleus typical of hematopoietic cells. CD31+ events were distinguished by one of two morphologically distinct patterns depending on cellular context: circulating endothelial-like cells (CECs; CK variable, positive or negative) display a larger, elongated or spindle-shaped cellular morphology with vimentin expression outlining the cell body, together with diffuse CD-channel positivity, whereas platelet coating on a CK+ tumor cell (scored as pc.CTC or pc.mes.CTC) presents as a punctate, peripheral, cell-surface-restricted CD signal ringing the cell, morphologically distinct from the diffuse, cell-body CD signal used to call CD45+ immune-like CTCs (im.CTC). Events with ambiguous morphology were classified as indeterminate rather than forced into a category. Cells were classified into phenotypic categories based on marker combinations and morphology, including epithelial CTCs, mesenchymal CTCs, hybrid phenotypes, leukocytes, and other atypical or indeterminate cells (Table 2). Cell counts were normalized to sample volume to enable comparisons across compartments; where CTC burden is instead expressed as a percentage, this denotes the proportion of all DAPI+ nucleated cells classified as CTCs, and the two metrics (CTCs/mL and CTC%) should not be conflated, since total sample cellularity varied substantially across compartments.

In figure gating legends, D denotes DAPI positivity and the ‘|’ symbol denotes co-positivity for the markers listed (e.g., D|CK|V indicates DAPI+/CK+/VIM+). Legend entries are not identical across figure panels because they list only the phenotype categories actually observed in that patient’s sample: DAPI−/CK+ events, consistent with acellular, large extracellular-vesicle-like structures, were observed in Patients 1 and 2; Patient 3 additionally showed several further DAPI−/CK+ vesicle-like categories (including DAPI−/CK+/VIM+); and no such events were observed in Patients 4 or 5, so these categories do not appear in those panels’ legends. The enrichment-free fWSI/HDSCA platform used here has been analytically validated in prior work, including spike-in linearity experiments demonstrating strong agreement between expected and observed cell recovery across approximately 10–300 spiked cancer cells per slide (R^2^ = 0.9997), along with split-sample robustness testing across processing batches [11]. Dedicated positive/negative control spike-ins were not repeated for the present cohort, and formal false-positive/false-negative rates for the current classification scheme, including the morphology-based CD45/CD31 criteria above, are not available for this specific cohort (see Limitations).

### 2.5. Single-Cell Genomic Analysis

To assess genomic heterogeneity across compartments and evaluate whether CTC populations within the same patient shared common clonal ancestry, single-cell copy number variation (CNV) profiling was performed on select cells isolated from PB and CSF samples.

Cells of interest were relocated on the imaging slides and re-imaged at 40× magnification for confirmatory morphometric review. As a standard procedure aimed at minimizing DNA fragmentation, cells were extracted within 5 days of the initial staining procedure. Individual cells were extracted using an Eppendorf TransferMan NK2 micromanipulator equipped with a 25° jagged micropipette (Piezo Drill Tip ES, Eppendorf, Hamburg, Germany) by applying fluid suction. Each captured cell was rinsed with phosphate-buffered saline (PBS) and deposited into a 0.2 mL PCR tube containing 2 μL of lysis buffer (200 mM KOH; 50 mM DTT). Tubes were immediately frozen at −80 °C and transferred on dry ice to the sequencing laboratory, where whole-genome amplification (WGA) was performed using the WGA4 Genomeplex Single Cell Whole Genome Amplification Kit (Sigma-Aldrich, St. Louis, MO, USA), followed by library construction and low-pass whole-genome sequencing [12,21].

Single-cell CNV profiling was performed by low-pass whole-genome sequencing. Sequencing reads were aligned to the human reference genome (hg19) using paired-end reads. Read depth was calculated in approximately 250 kb genomic bins, and the ratio to the median bin count was computed for each cell to generate a log_2_ copy number ratio profile. Segmentation was applied to define regions of consistent copy number. Hierarchical clustering was performed using Ward’s linkage with Manhattan distance, and heatmaps were generated with chromosomal regions on the *y*-axis and individual cells on the *x*-axis. Copy number gains are displayed in red and losses in blue; color intensity reflects the magnitude of deviation from copy-neutral thresholds (upper: 1.25; lower: 0.75).

### 2.6. Data Analysis

Analyses focused on descriptive comparisons of cellularity and rare cell incidence across anatomical compartments and timepoints within individual patients. Given the limited cohort size and heterogeneity of clinical contexts, no formal hypothesis testing was performed.

### 2.7. Sample Quality Control

Given the post-mortem origin of the majority of samples, potential pre-analytical confounders were assessed systematically (Table 1). Relevant factors include post-mortem interval (all samples collected within the 6–8 h Legacy program window), WBC count at processing, cold-storage duration prior to processing, and CSF amplification success rate. Where sample quality concerns were identified—specifically overnight 4 °C storage of Patient 4 CSF prior to shipment, and amplification failure in Patient 3 CSF—findings from those samples are reported with explicit caveats and are not used to draw biological conclusions.

A schematic of the sampled anatomical compartments is provided in Supplementary Figure S1 to orient the compartment terminology used throughout.

## 3. Results

Circulating rare cells were classified by immunophenotype into the following categories: epithelial CTCs (epi.CTC; CK+/VIM−/CD−), mesenchymal CTCs (mes.CTC; CK+/VIM+/CD−), immune-like CTCs (im.CTC; CK+/CD45+), platelet-coated CTCs (pc.CTC; CK+/CD31+), and their mesenchymal counterpart, platelet-coated mesenchymal CTCs (pc.mes.CTC; CK+/VIM+/CD31+). Additional non-tumor rare cell populations were also cataloged, including cells classified as circulating endothelial cell-like (CEC; DAPI+/CK−/VIM+/CD31+) and cancer-associated fibroblast-like (cCAF; DAPI+/CK−/VIM+/CD−) phenotypes. These classification labels reflect immunophenotypic pattern matching and do not assert a definitive cell identity; both CECs and cCAFs represent heterogeneous populations that may include other cell types with similar staining profiles. Full phenotypic criteria are summarized in Table 2.

### 3.1. Patient and Sample Characteristics

Five patients with primary breast cancer that eventually metastasized to LMD were enrolled (Table 3; Supplementary Table S1). Tumor subtypes included two ER+/PR+/HER2− invasive lobular carcinomas, one triple-negative invasive ductal carcinoma, one ER+/HER2+ infiltrating ductal carcinoma (Patient 3), and one ER−/HER2+ infiltrating ductal carcinoma (Patient 4). Patients 1 and 3 had samples taken both ante- and post-mortem; all other patients had post-mortem-only sampling. Disease courses ranged from treated LMD with periods of remission to rapidly progressive, untreated CNS disease. Across patients, a total of 23 ante-mortem and post-mortem PB (*n* = 11) and CSF (*n* = 12) samples were analyzed, with post-mortem collections providing access to multiple blood and CSF compartments. A brief overview of each case is presented prior to general observations across the cohort.

#### Overview: Cross-Patient Patterns, Single-Patient Observations, and Technical Considerations

Given the small, clinically heterogeneous cohort (*n* = 5), we distinguish three categories of findings below and in the patient-level narratives that follow, summarized across patients in Table 4. (i) Patterns observed in more than one patient, which we consider the most robust descriptive signal in this dataset: higher total rare cell counts in arterial than paired venous post-mortem blood, observed in both patients with paired arterial–venous PB (Patients 2 and 3); and concordant CNV profiles among CSF-derived CTCs within a given patient, observed in the three patients with successful CSF single-cell amplification (Patients 1, 2, and 5). (ii) Single-patient observations, reported descriptively and not generalized to the cohort: the right-greater-than-left ventricular CTC gradient (Patient 1 only, the sole patient with sampling across all four ventricular/cisternal sites), and the association between intrathecal therapy and lower CSF CTC burden (based on two treated patients with differing outcomes and at least one plausible technical confound; see Limitations). (iii) Technical observations attributable to sample handling rather than underlying biology, detailed as QC flags in Table 1: delayed processing of Patient 4 CSF, amplification failure in Patient 3 CSF, and low WBC count in Patient 2 venous PB. Given the cohort size, no formal statistical testing was performed for any comparison; all observations below should be read as descriptive and hypothesis-generating rather than as validated conclusions.

### 3.2. CSF: Cellularity and Regional Heterogeneity

CSF samples exhibited striking variability in both total cellularity and CTC frequency across anatomical compartments within the same patient. Ventricular CSF samples generally demonstrated higher CTC fractions than distal compartments such as the foramen magnum, based on the patients with CSF sampled at more than one site along this axis. Importantly, differences in CTC incidence were not strictly correlated with total nucleated cell counts, indicating true compartmental differences rather than sampling depth alone.

Patients with untreated or aggressive CNS disease demonstrated extremely high CSF CTC fractions, often comprising a substantial proportion of all nucleated cells. Both epithelial and mesenchymal CTC phenotypes were observed, with marked morphological heterogeneity. In contrast, the two patients receiving intrathecal therapy (Patients 1 and 3) each showed low or absent CSF CTCs across sampled compartments. We interpret this association cautiously: it rests on only two treated patients, Patient 1 showed persistent right-ventricular CTCs despite therapy (see below), and, as detailed in the Patient 4 narrative, a third patient who did not achieve CNS control on intrathecal therapy also had a low CSF CTC value that is more plausibly explained by antigen degradation in a delayed-processing sample than by treatment effect. We therefore do not interpret these data as establishing a general link between intrathecal therapy and CSF CTC clearance. Across CSF samples, abundant leukocytes were observed, frequently in close proximity to tumor cells. Mesenchymal and hybrid CTC phenotypes were detected at a higher incidence in patients with aggressive or uncontrolled disease (Figure 1), whereas epithelial phenotypes predominated in treated or stable cases. Sample quality metrics relevant to CSF interpretation are summarized in Table 1.

Genomic profiling of the isolated cells further elucidated this compartmental divergence. While the PB contained a mixture of circulating rare cell phenotypes that remained predominantly genomically stable with few altered CTCs, all the CTCs isolated from the CSF (all CK+ cells with successful amplification) exhibited extensive chromosomal aberrations. This finding indicates that the epithelial CTCs within the CSF compartment are not sporadic shedding events but represent a genomically evolved cell population with convergent chromosomal architecture—consistent with, though not formally proving, clonal dominance within the leptomeningeal niche, and we cannot exclude that sampling bias (a small number of cells drawn from a single compartment) or shared technical amplification effects contribute to the observed concordance. The concordance of CNV profiles across phenotypically distinct CTC subtypes within the same CSF compartment (as seen in Patient 2) further supports a shared genomic origin of morphologically diverse CSF CTCs.

### 3.3. Peripheral Blood: Arterial-Venous Differences and CTC Plasticity

The PB compartment exhibited significant cellular plasticity, characterized by a diverse spectrum of circulating rare cells and varying degrees of genomic stability. Single-cell phenotyping and corresponding CNV analysis (Figure 2) revealed distinct CTC subtypes, including epi.CTCs (CK+/VIM−), mes.CTCs (CK+/VIM+), im.CTCs (CK+/CD45+) and platelet-coated (pc).CTCs (CK+/CD31+). CTC subtypes displayed complex genomic alterations, including recurrent chromosomal gains and losses, confirming their malignant origin and reflecting the high degree of intratumoral heterogeneity within the blood.

In contrast to the clonal alterations observed in CTCs, other rare cell populations associated with the tumor microenvironment (TME)—including cells classified as CEC-like (DAPI+/CK−/VIM+/CD31+), cCAF-like (DAPI+/CK−/VIM+/CD−), and megakaryocyte-associated phenotypes (Figure 2B)—remained genomically neutral or minimally altered. This genomic distinction underscores that the detected cellular plasticity is a specific feature of the malignant CTC population, rather than reflecting a general instability of non-tumor rare cell populations.

In patients with longitudinal ante-mortem sampling, peripheral blood CTC burden increased over time in association with disease progression. In contrast, patients with controlled systemic disease or extensive intrathecal treatment demonstrated lower peripheral CTC incidence, despite high overall cellularity. For two patients (Patients 2 and 3, the only two patients in this cohort with both arterial and venous PB collected at the same, post-mortem, timepoint; Patient 1’s arterial and venous PB were collected at different timepoints, ante-mortem venous and post-mortem arterial only, and Patients 4 and 5 had post-mortem arterial PB only, without a paired venous draw) we were able to compare the post-mortem arterial and venous blood compartments. Arterial blood showed higher total rare cell counts than venous blood in both patients. In terms of CK-positive CTC incidence specifically, arterial exceeded venous in Patient 2 (1425.73 vs. undetectable epi.CTC/mL), while Patient 3 showed the reverse for this specific metric (69.82 arterial vs. 88.40 venous epi.CTC/mL), despite arterial still showing higher total rare cell counts in that patient. We therefore distinguish these two metrics: arterial blood showed higher total rare cell counts in both paired comparisons available, but higher CK-positive CTC incidence in only one of the two.

This pattern was most pronounced in patients with active systemic disease and minimal prior treatment, where arterial samples contained a high incidence of epi.CTCs, while paired venous samples showed markedly reduced or absent CTC detection. This suggests a rapid clearance or phenotypic transition of CTCs as they traverse the capillary beds, though this specific mechanistic interpretation is not directly tested by the present data. Rare CK-positive cells with atypical morphology were also observed in select ventricular samples, suggesting possible contributions from CNS-resident epithelial-like populations or a high degree of compartmentalized plasticity during CNS dissemination.

### 3.4. Patient 1

Patient 1 was a patient with ER+/HER2− (ERBB2 L755S is a mutation, not HER2 amplification/overexpression; HER2 was negative by testing, see Table 3) breast cancer exhibiting systemic metastases including liver and skin, as well as brain involvement, with suspected LMD alongside a benign meningioma. At initial diagnosis in 2017, the primary tumor was ER+/HER2− by standard tissue immunohistochemistry/FISH testing. A Guardant360 circulating-tumor-DNA panel obtained in 2020 subsequently identified several ERBB2 (HER2) mutations (L755S, 10.5% variant allele frequency; D769Y, 0.4%; L786V, 0.4%), together with PIK3CA H1047R (11.8% VAF), high-level CCND1 amplification, and PTEN F257L (2.5% VAF); these are HER2-mutant, not HER2-amplified, findings, and are the basis for the ERBB2 alterations listed in Table 3 rather than a change in tissue HER2 status. The patient was diagnosed with LMD in September 2021 by brain MRI and positive CSF cytology. An Ommaya reservoir was placed in October 2021, and the patient received intrathecal topotecan therapy from April to May. Ante-mortem peripheral blood (PB) collections in January and March showed an increase in epi.CTCs from 19.16 to 45.86 CTCs/mL (Figure 3A), suggesting progressive systemic disease.

Post-mortem analysis in July included arterial PB and CSF from multiple compartments (left and right ventricles, fourth ventricle, foramen magnum). Arterial PB contained 27.68 epi.CTCs/mL. CSF CTCs were highest in the right ventricle (11.4%), followed by left ventricle (6.4%), fourth ventricle (4.5%), and lowest at the foramen magnum (0.6%), indicating compartmental variation (Figure 3B); this proximal-to-distal, right-greater-than-left pattern was assessed in this patient alone, the only case with CSF sampled across all four ventricular/cisternal sites, and is reported as a single-patient observation, not a cohort-level finding. The asymmetric right > left ventricular CTC gradient may reflect anatomic proximity of the dominant tumor focus to the right ventricular system, or localized tumor-CSF interface density—consistent with the rostrocaudal dilution model [15,16]. The persistence of CTCs in the right ventricle despite intrathecal topotecan therapy may indicate localized high tumor burden in that compartment, limited drug penetration, or a combination of both.

A total of 19 single cells were isolated at the post-mortem timepoint for downstream genomic analysis, comprising 10 cells from post-mortem arterial PB and 9 cells from CSF collected via the Ommaya reservoir (right lateral ventricle) (Figure 3C). CNV profiling revealed distinct patterns between compartments, with CSF-derived cells exhibiting highly concordant genomic alterations. All CSF-derived epi.CTCs demonstrated a uniform CNV profile, consistent with a clonal population, though, as noted above, sampling of a limited number of cells from a single compartment cannot exclude the possibility that this apparent concordance partly reflects sampling bias or shared technical amplification effects rather than biology alone. In contrast, PB-derived cells showed greater heterogeneity, with most cells lacking clear tumor-associated CNV patterns. However, 1 epi.CTC identified in arterial PB displayed genomic alterations highly similar to those observed in CSF CTCs, suggesting a potential shared clonal origin and supporting the hypothesis of tumor cell trafficking between systemic circulation and the CSF compartment.

### 3.5. Patient 2

This patient was diagnosed with triple-negative breast cancer in April 2021 and showed brain metastases and LMD involvement a year later. No Ommaya reservoir was placed; the patient did not receive intrathecal treatment. No ante-mortem CSF or blood samples were collected. Post-mortem analysis revealed marked compartmental differences in CTCs: arterial peripheral blood contained 1425.73 epi.CTCs/mL with a heterogeneous population of rare cells, while venous blood showed no detectable epi.CTCs, though low WBC counts raise potential concerns regarding sample integrity (Figure 4A). CSF collected from left and right ventricles exhibited similar high cellularity, containing both epithelial and mesenchymal CTCs, indicative of aggressive, uncontrolled leptomeningeal disease (Figure 4B).

A total of 32 single cells were isolated for genomic analysis, including 24 cells from post-mortem arterial PB and 8 cells from post-mortem CSF collected from the left ventricle (Figure 4C). CNV profiling demonstrated clear compartment-specific differences, with CSF-derived cells showing highly concordant genomic patterns. All CSF-derived CTCs, encompassing both epi.CTC and mes.CTC phenotypes, exhibited a consistent CNV profile indicative of a convergent genomic architecture (as above, based on a small number of cells from one compartment). In contrast, PB-derived cells displayed substantial heterogeneity. Among these, 2 CTCs (mes.CTC and pc.mes.CTC) shared highly similar genomic profiles with the CSF clonal population, suggesting a common clonal origin and possible bidirectional trafficking between the systemic circulation and central nervous system.

### 3.6. Patient 3

This patient had ER+/HER2+ breast cancer with prior brain and bone metastases and suspected LMD but was in clinical remission at the time of analysis. An Ommaya reservoir was placed in November 2022 for intrathecal Herceptin therapy. Ante-mortem collections included one PB sample showing 2.98 epi.CTCs/mL and two ante-mortem CSF samples, the second of which showed 0% CTCs. At the separate post-mortem timepoint, arterial and venous PB CTC counts were both high (69.82 and 88.40 epi.CTCs/mL, respectively), venous exceeded arterial for this CK-positive CTC metric, the one exception in the cohort to the arterial > venous pattern described above (Section 3.3), for reasons not evident from the available clinical data, while the separate post-mortem CSF samples from the Ommaya and foramen magnum contained minimal CTCs (0.05–0.1%), consistent with controlled CNS disease at that later timepoint (Figure 5A,B).

A total of 17 cells were isolated from post-mortem arterial PB for genomic analyses. Copy number profiling (Figure 5C) revealed sparse genomic alterations. Overall, the genomic landscape did not reveal evidence of a dominant clonal population, consistent with the absence of widespread genomic aberrations among the analyzed cells. Single-cell genomic amplification failed for all isolated post-mortem (Ommaya/foramen magnum) CSF cells; this most likely reflects the age of the sample at processing combined with the very low number of cells recovered (*n* = 3), rather than a broader sampling issue, though disease control at this timepoint cannot be excluded as a contributing factor to the low cell yield itself.

### 3.7. Patient 4

A patient with HER2-positive breast cancer presented with extensive metastases to bone, brain (including LMD), and liver, exhibiting a large CNS tumor burden with some necrotic lesions consistent with prior cranial radiotherapy. The patient received intrathecal Herceptin via lumbar puncture in April and May 2024; however, therapy was insufficient to control CNS disease, as evidenced by seizures, blindness, and inflammatory MRI flares. No Ommaya reservoir was placed. Post-mortem evaluation showed low CTC incidence in PB (3.4 and 9.4 epi.CTCs/mL; Figure 6A) and CSF (0.02%), despite the foramen magnum CSF sample being highly cellular (Figure 6B); technical factors—specifically overnight storage at 4 °C prior to shipment—likely contributed to antigen degradation and reduced CK detection (see Table 1, QC flags); as a result, this low CSF CTC value cannot be taken as evidence of treatment response despite two months of intrathecal therapy, and should not be used to support a general link between intrathecal therapy and CSF CTC clearance (see also Section 3.2). No cells were isolated for genomic analyses.

### 3.8. Patient 5

A patient with ER+/PR+/HER2− invasive lobular carcinoma presented with bone, liver, and peritoneal metastases. The patient had received prior hormonal therapy but no intrathecal therapy prior to death. No Ommaya reservoir was placed, and no ante-mortem CSF samples were available. Post-mortem evaluation revealed high CTC counts in blood (398.2 epi.CTCs/mL; Figure 7A) and in CSF collected from the foramen magnum (7.18% CTCs; Figure 7B). Both epithelial CTCs and mesenchymal CTCs were detected in the CSF. Brain imaging prior to death was reportedly normal, but autopsy confirmed extensive brain metastases, highlighting the utility of post-mortem multi-compartment liquid biopsy for uncovering occult CNS disease.

A total of 22 cells were isolated for single-cell genomic analyses, comprising 18 cells from the post-mortem arterial PB and 4 cells from the post-mortem CSF collected at the foramen magnum. Copy number profiling (Figure 7C) revealed a stark divergence in genomic stability between compartments. While the cells isolated from the PB remained predominantly neutral with only sporadic, non-recurrent alterations, the cells isolated from the CSF exhibited extensive and highly concordant chromosomal aberrations, forming a distinct, homogeneous cluster consistent with expansion of a clonally convergent tumor cell population (*n* = 4 CSF cells; see the general caveat on sampling bias and technical amplification effects above). Specifically, all 4 CSF-derived cells displayed a recurrent pattern of large-scale amplification (most notably on chromosomes 1, 5, 12, and 20) alongside shared deletions on chromosome 10.

## 4. Discussion

This study demonstrates that the liquid biopsy landscape in LMD is highly compartmentalized, with significant genomic and phenotypic divergence observed between arterial and venous blood, as well as across distinct anatomical regions of the CSF. Using an enrichment-free fWSI approach to characterize single cells in the liquid biopsy, we show that rare tumor-associated cells are unevenly distributed across circulatory and CNS compartments, reflecting underlying disease biology, treatment exposure, and clinical trajectory.

A key finding of this study is the stark contrast in clonal architecture between systemic and CNS compartments. In the CSF, 100% of isolated epithelial CTCs exhibited extensive chromosomal aberrations. This limited genomic heterogeneity in the CSF is consistent with our own prior single-cell characterization of CSF CTCs in leptomeningeal disease [20], and broadly with reports on CTC detection in CSF and its prognostic significance [22,23] and with evidence of branched clonal evolution in brain metastases more generally [24]; we note that [22,23] primarily address detection and outcome rather than clonal architecture specifically, so we regard [20] as the most directly comparable clonality-focused precedent. This suggests that the leptomeningeal space acts as a selective niche where a specific, genomically evolved subclone thrives. In contrast, the peripheral blood displayed a broader spectrum of genomic stability and phenotypic variation, consistent with the extensive heterogeneity typically observed in systemic CTC populations [8,9].

Our data highlight significant cellular plasticity in the peripheral blood compartment. The enrichment of mesenchymal and hybrid CTC phenotypes in untreated or aggressive cases suggests that cellular plasticity is associated with terminal disease progression and treatment resistance [25]. The genomic neutrality of TME-associated non-tumor cells (CECs, cCAFs, megakaryocytes) confirms that this plasticity is a specific feature of the malignant cell population rather than a technical artifact. The higher incidence of total rare cells, and in one of two paired patients CK-positive CTCs specifically, in arterial compared to venous blood is consistent with a model in which arterial blood captures tumor cells proximal to their vascular entry from tumor sources, prior to peripheral capillary sequestration, immune clearance, or phenotypic transition during tissue transit, consistent with in vivo evidence that hemodynamic forces govern the arrest, adhesion, and extravasation of circulating tumor cells within capillary beds [26]. We note the one exception among paired patients (Patient 3), where venous CK-positive CTC incidence exceeded arterial.

Within the CNS, the pronounced heterogeneity in CSF cellularity challenges the notion of CSF as a uniform diagnostic compartment. Differences in CTC burden across ventricular and cisternal locations likely reflect a combination of tumor proximity, CSF flow dynamics, and local microenvironmental factors, based primarily on the one patient (Patient 1) sampled across all four sites along this axis. These findings suggest that single-site CSF sampling may underestimate disease burden and that regionally informed sampling strategies could improve diagnostic yield. Further, treatment exposure emerged as a candidate modifier of liquid biopsy findings: the two patients receiving intrathecal therapy in this cohort showed lower CSF CTC incidence, though one (Patient 1) retained persistent right-ventricular CTCs, and a third patient’s low CSF CTC value (Patient 4) is confounded by a technical concern related to antigen-degradation. We regard the relationship between intrathecal therapy and CSF CTC clearance as a hypothesis for prospective testing rather than an established finding.

This study has several important limitations. First, the cohort of five patients is small and clinically heterogeneous; this work is explicitly hypothesis-generating and not powered for statistical inference. Formal statistical testing and outcome correlations will require larger, prospectively designed studies. Second, post-mortem confounders—including agonal physiology, post-mortem interval effects on cell integrity, and compartment-specific collection conditions—are addressed systematically in Table 1 but cannot be fully excluded. Third, CNV profiling from 4 to 9 cells per compartment cannot formally establish clonal dominance; this requires larger cell numbers or orthogonal SNV-level approaches. The apparent concordance among CSF-derived CTCs should also be interpreted with some caution because (a) all CSF cells within a given patient were drawn from the same compartment and timepoint, so sampling bias, a small number of cells reflecting a locally dominant subpopulation rather than the full extent of intra-tumoral diversity, cannot be excluded, and (b) low-input whole-genome amplification can introduce recurrent, non-biological copy-number artifacts that could modestly inflate apparent similarity among cells co-processed in a batch; while we consider this a minor contributor relative to biological clonal concordance, the present data cannot fully rule out a technical contribution. The fWSI workflow’s analytical performance is well established, with prior validation including spike-in linearity across approximately 10–300 cells per slide (R^2^ = 0.9997) and split-sample robustness testing [11]. In this cohort, concordant molecular (CNV) profiling of individually isolated cells further supported true-positive CTC/CEC calls, though phenotype assignments were not corroborated by orthogonal proteomic methods, so protein-level false-positive/false-negative rates remain undetermined for this population. Relatedly, the morphology-based criteria used to resolve CD45+ versus CD31+ identity within the combined CD channel have not been confirmed at the molecular level in this cohort, so phenotype-specific misclassification could contribute to the compartmental differences reported here. Fourth, the absence of paired cfDNA/ctDNA or soluble proteomics data from the same samples limits multi-analyte comparison. Finally, as a warm autopsy program, this study is limited to patients at the very end of their disease course, providing only a terminal snapshot rather than longitudinal LMD evolution.

In summary, our findings offer a regionally resolved view of liquid biopsy in leptomeningeal disease and underscore the potential value of enrichment-free, single-cell approaches for capturing the complexity of tumor dissemination across systemic and CNS compartments; given the small cohort, we present the following as hypotheses arising from this dataset rather than established conclusions. CSF may not be a uniform diagnostic compartment: in the one patient sampled across the full ventricular-to-cisternal axis, CTC burden decreased along that axis in a pattern consistent with CSF flow dynamics and tumor proximity, a hypothesis warranting testing in patients with comparably complete multi-site sampling. Within CSF, convergent CNV architecture is consistent with, though does not formally establish, clonal expansion of a CNS-adapted tumor cell population, in contrast to the broader genomic heterogeneity of systemic CTCs. Intrathecal therapy was associated with lower CSF CTC burden in the two treated patients in this cohort, but this association rests on limited numbers with at least one technical confound and should be tested prospectively before it is used to guide monitoring. These findings support further investigation of regionally informed sampling strategies and CTC-based pharmacodynamic monitoring. They also lead to the hypothesis that the leptomeningeal space functions as a selective niche sustaining a genomically evolved tumor subclone during advanced breast cancer dissemination.

## 5. Conclusions

The following are offered as hypotheses generated by this exploratory cohort, to be tested in larger, prospectively designed studies before they inform practice. Current single-site CSF sampling may underestimate disease burden because tumor-associated signals appeared unevenly distributed across CSF compartments in our data, consistent with, though not a direct test of, the reported 40–50% false-negative rate of a single lumbar puncture [6]. Anatomically guided sampling (ventricular access for intracranial disease, lumbar puncture for spinal disease) combined with enrichment-free CTC detection is a candidate strategy for improving diagnostic sensitivity that warrants prospective evaluation. CTC burden in CSF may reflect both treatment response and resistance, raising the possibility of CTC profiling as a pharmacodynamic and resistance biomarker. Identification of mesenchymal and hybrid phenotypes may help flag higher-risk patients, pending validation in larger cohorts. The frequent co-detection of tumor and immune cells in CSF points to a possible approach for monitoring patients receiving CNS-directed immunotherapy. Finally, in the two patients with paired post-mortem arterial and venous PB in this cohort, arterial blood captured higher total rare cell counts in both and higher CK-positive CTC incidence in one, a preliminary finding, if confirmed in more patients, with implications for liquid biopsy protocol standardization.

## Figures and Tables

**Figure 1:**
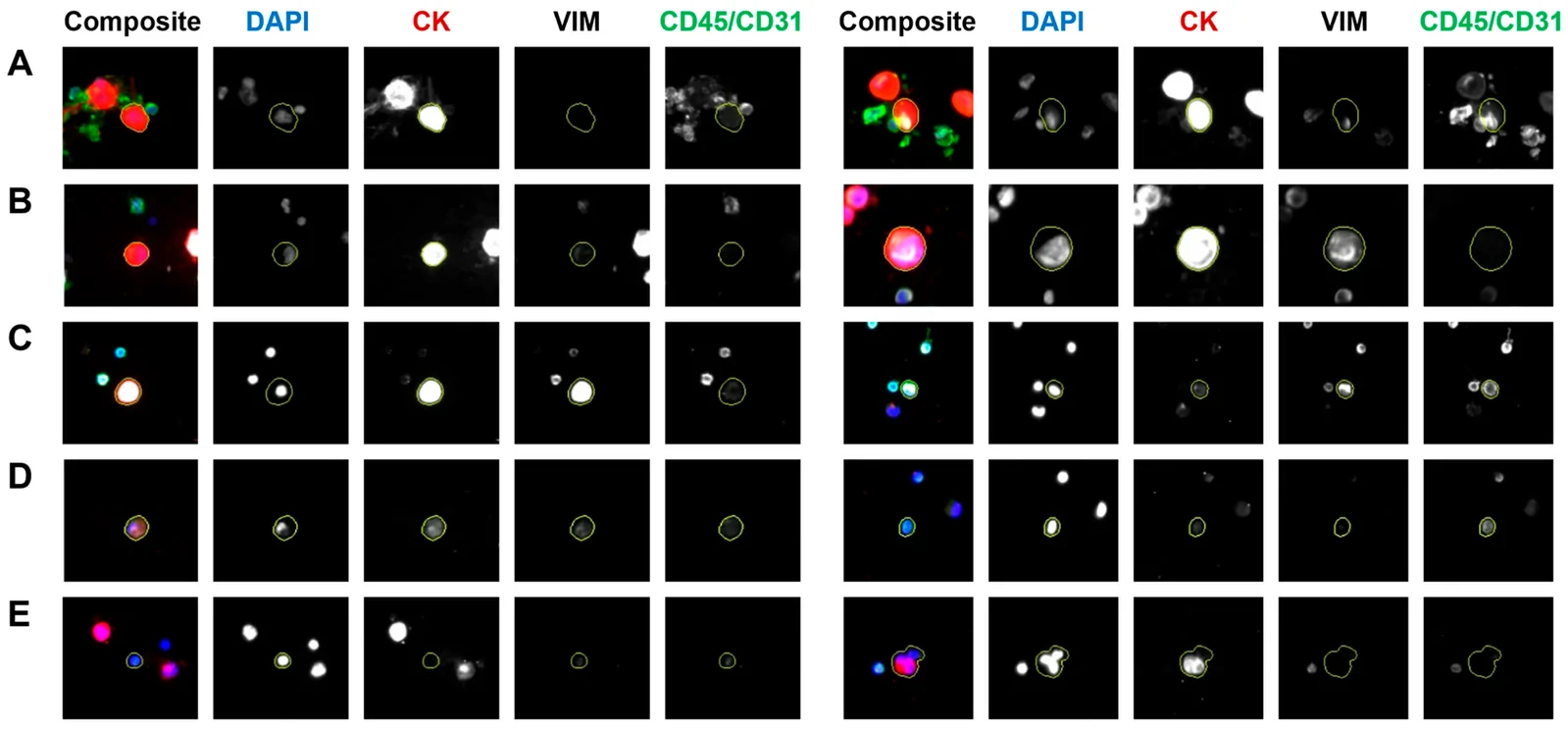
Representative circulating cell gallery from CSF samples. Images taken at 100× magnification. Composite immunofluorescence images show individual cellular events across patients ((**A**–**E**), two examples per row), identified by combinations of fluorescent markers. Each cell is displayed across five channels: composite overlay, DAPI (nuclear DNA, blue), cytokeratin (Alexa Fluor 555, red), vimentin (Alexa Fluor 488, white), and CD45/CD31 (Alexa Fluor 647, green). Cell outlines indicated by yellow contours in the DAPI channel. Rows correspond to patients: (**A**) Patient 1, (**B**) Patient 2, (**C**) Patient 3, (**D**) Patient 4, (**E**) Patient 5.

**Figure 2:**
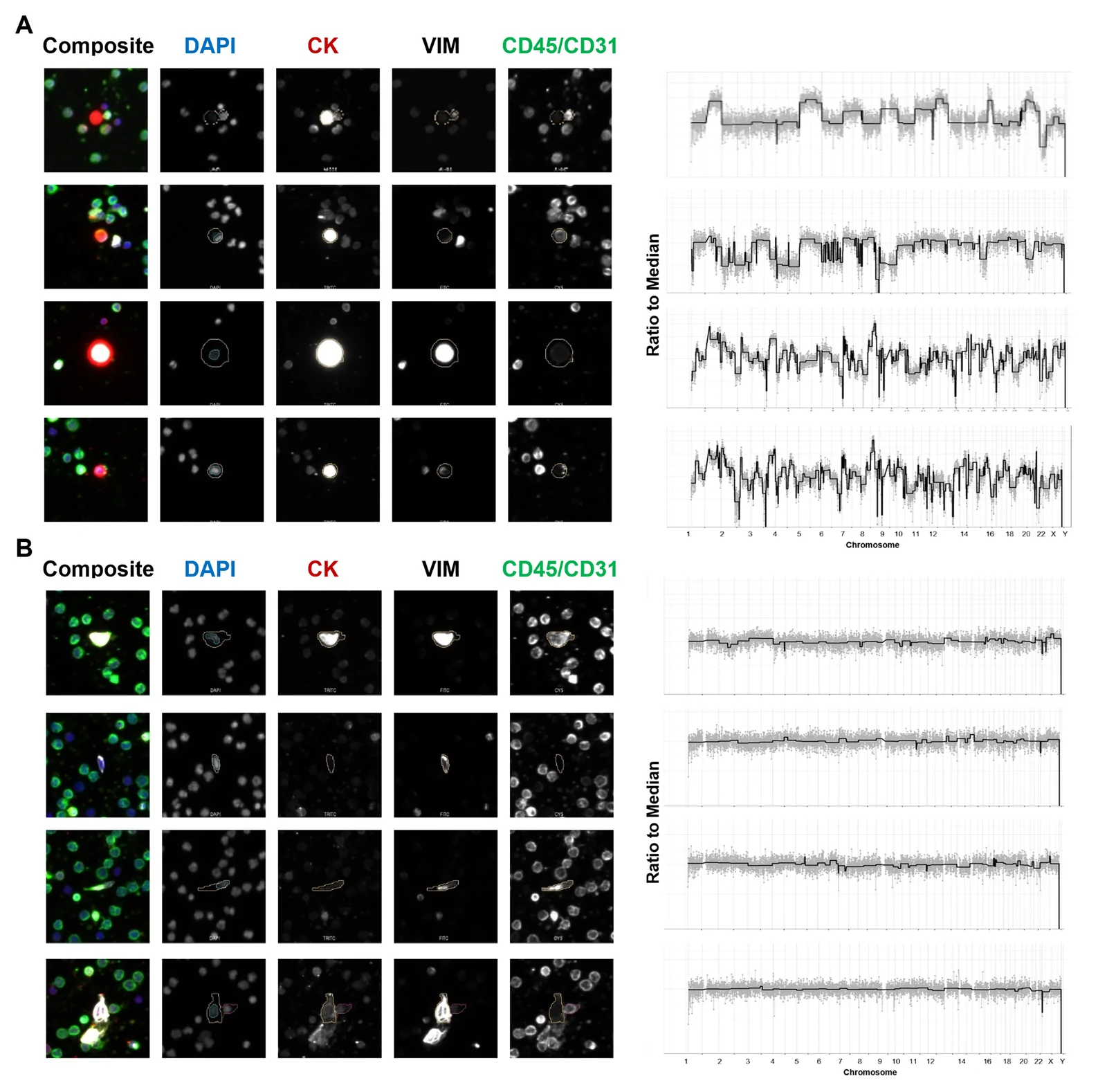
Circulating rare cells detected in the blood. (**A**) CTC subtypes with single cell genomic profile. (**B**) Other rare cells associated with TME. For each cell, the left panel displays the composite fluorescence image alongside individual single-channel images taken at 100× magnification: DAPI (nuclear DNA, blue), pan-cytokeratin (CK, red), vimentin (VIM, green), and CD45/CD31 (white). The right panel shows the corresponding single-cell copy number profile, displayed as the log_2_ ratio of observed-to-expected read depth (ratio to median) across the genome in 500 kb bins.

**Figure 3:**
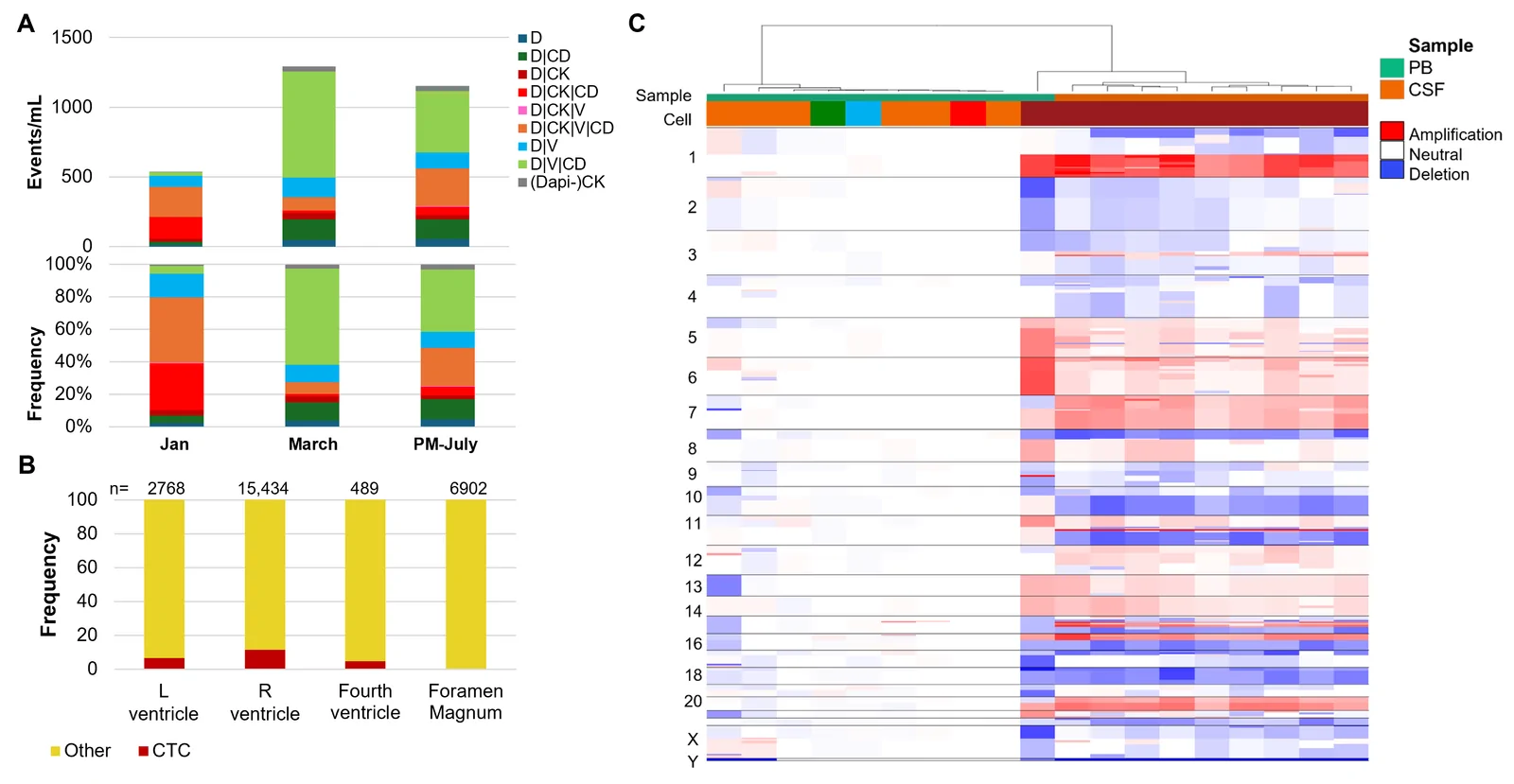
Patient 1 liquid biopsy analyses. (**A**) Counts (cell/mL) and frequency of rare cells found in the PB. (**B**) Frequency of all cells identified in the CSF. Cellular classifications are based on the expression profile of biomarkers (CK, VIM, CD) in the four fluorescent channels (DAPI, Alexa Fluor 488, Alexa Fluor 555, Alexa Fluor 647), indicating positive expression relative to the background level (see also the gating-legend definition, D = DAPI, ‘|’ = co-positivity, in Section 2.4). (**C**) Heatmap and phylogenetic tree of single-cell CNAs across the entire population of cells isolated. Each column represents an individual cell and each row shows chromosomal regions. For each sample, segments are shown as colored rectangles, where colors indicate copy number changes: white for neutral, red for gains, and blue for losses. The color intensity reflects the difference between the copy number and set thresholds (upper limit 1.25, lower limit 0.75).

**Figure 4:**
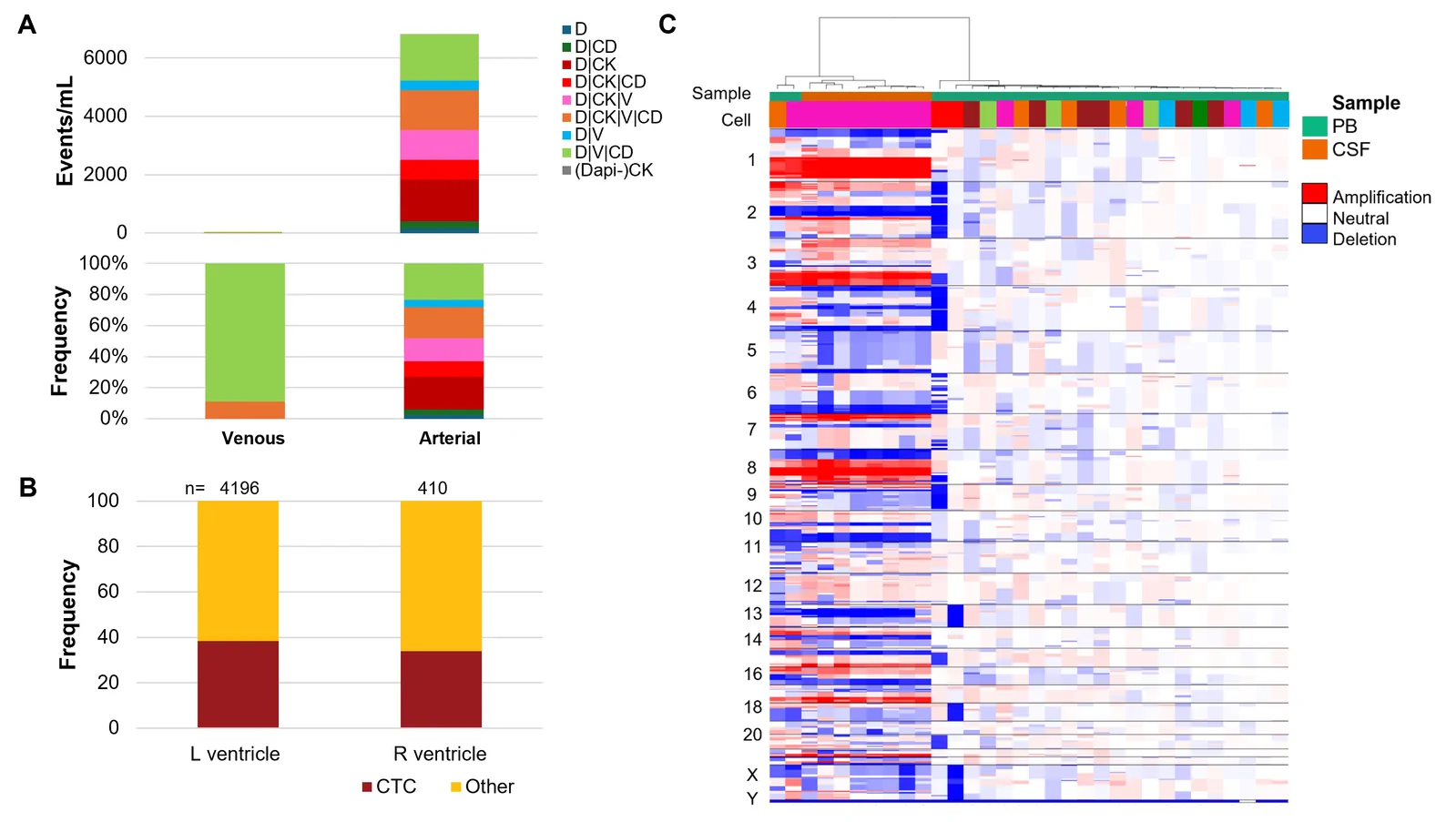
Patient 2 liquid biopsy analyses. (**A**) Counts (cell/mL) and frequency of rare cells found in the PB. (**B**) Frequency of all cells identified in the CSF. Cellular classifications are based on the expression profile of biomarkers (CK, VIM, CD) in the four fluorescent channels. (**C**) Heatmap and phylogenetic tree of single-cell CNAs across the entire population of cells isolated. Color scheme as in Figure 3.

**Figure 5:**
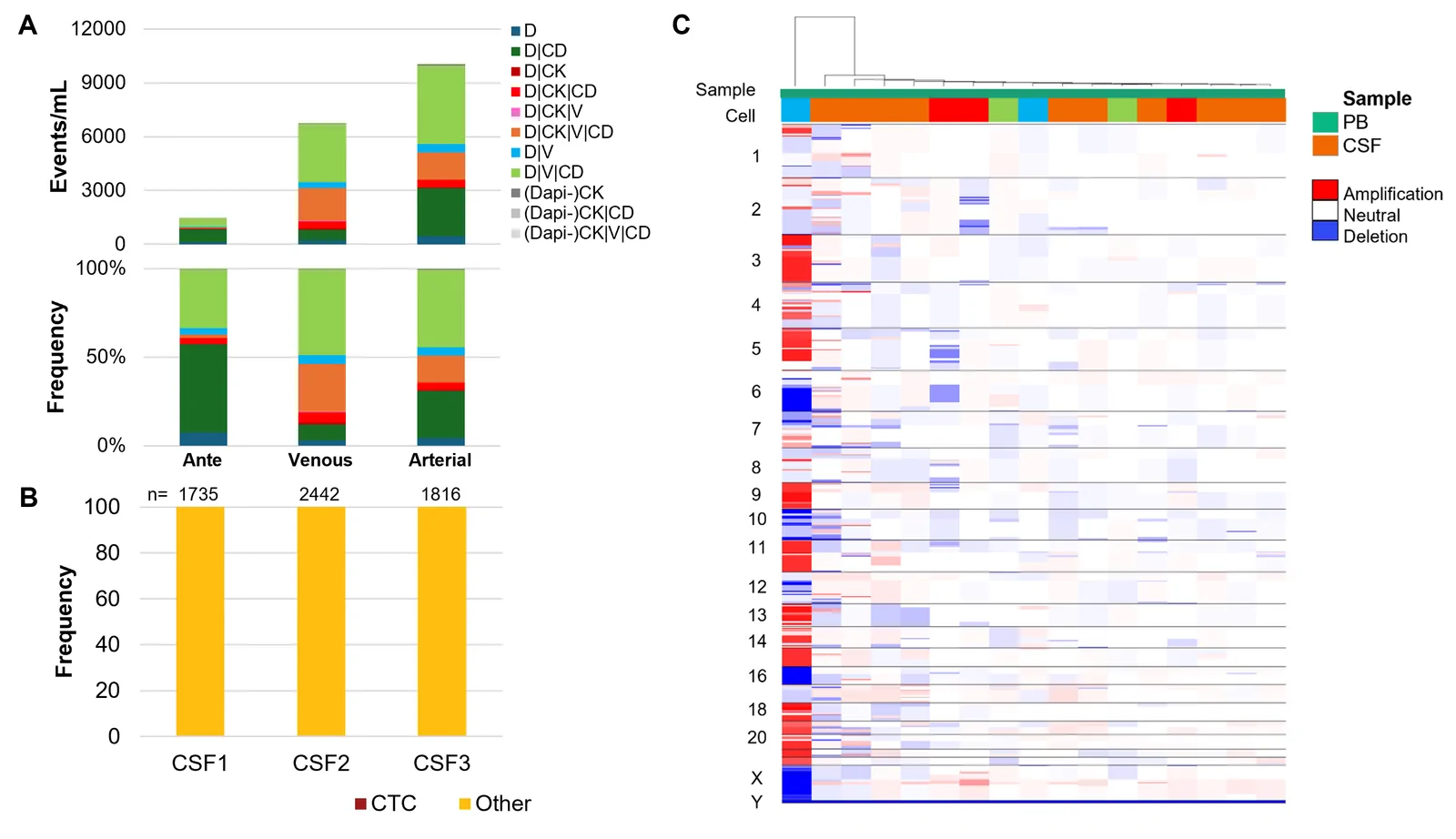
Patient 3 liquid biopsy analyses. (**A**) Counts (cell/mL) and frequency of rare cells found in the PB. (**B**) Frequency of all cells identified in the CSF. Cellular classifications are based on the expression profile of biomarkers (CK, VIM, CD) in the four fluorescent channels. (**C**) Heatmap and phylogenetic tree of single-cell CNAs across the entire population of cells isolated. Color scheme as in Figure 3.

**Figure 6:**
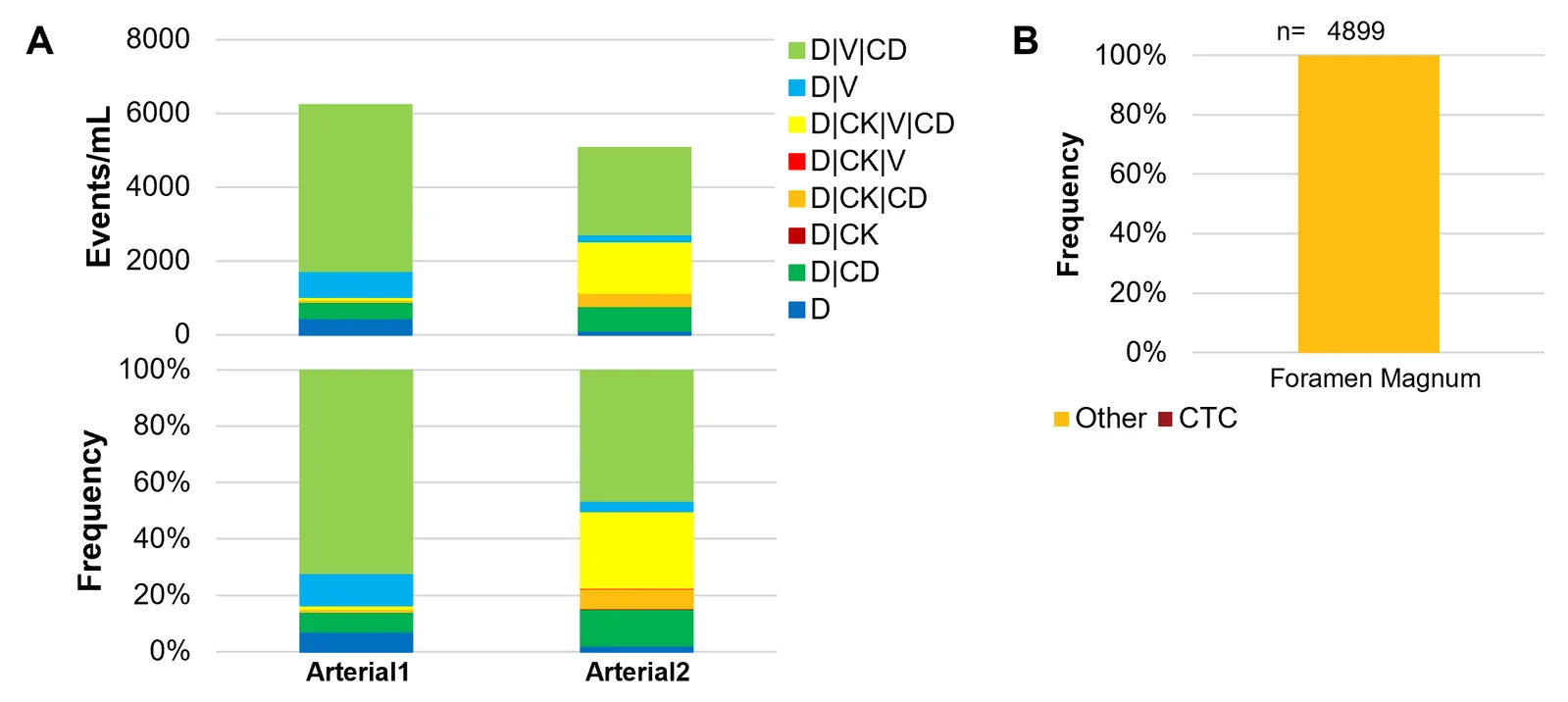
Patient 4 liquid biopsy analyses. (**A**) Counts (cell/mL) and frequency of rare cells found in the PB. (**B**) Frequency of all cells identified in the CSF. Cellular classifications are based on the expression profile of biomarkers (CK, VIM, CD) in the four fluorescent channels. No single-cell genomics were conducted.

**Figure 7:**
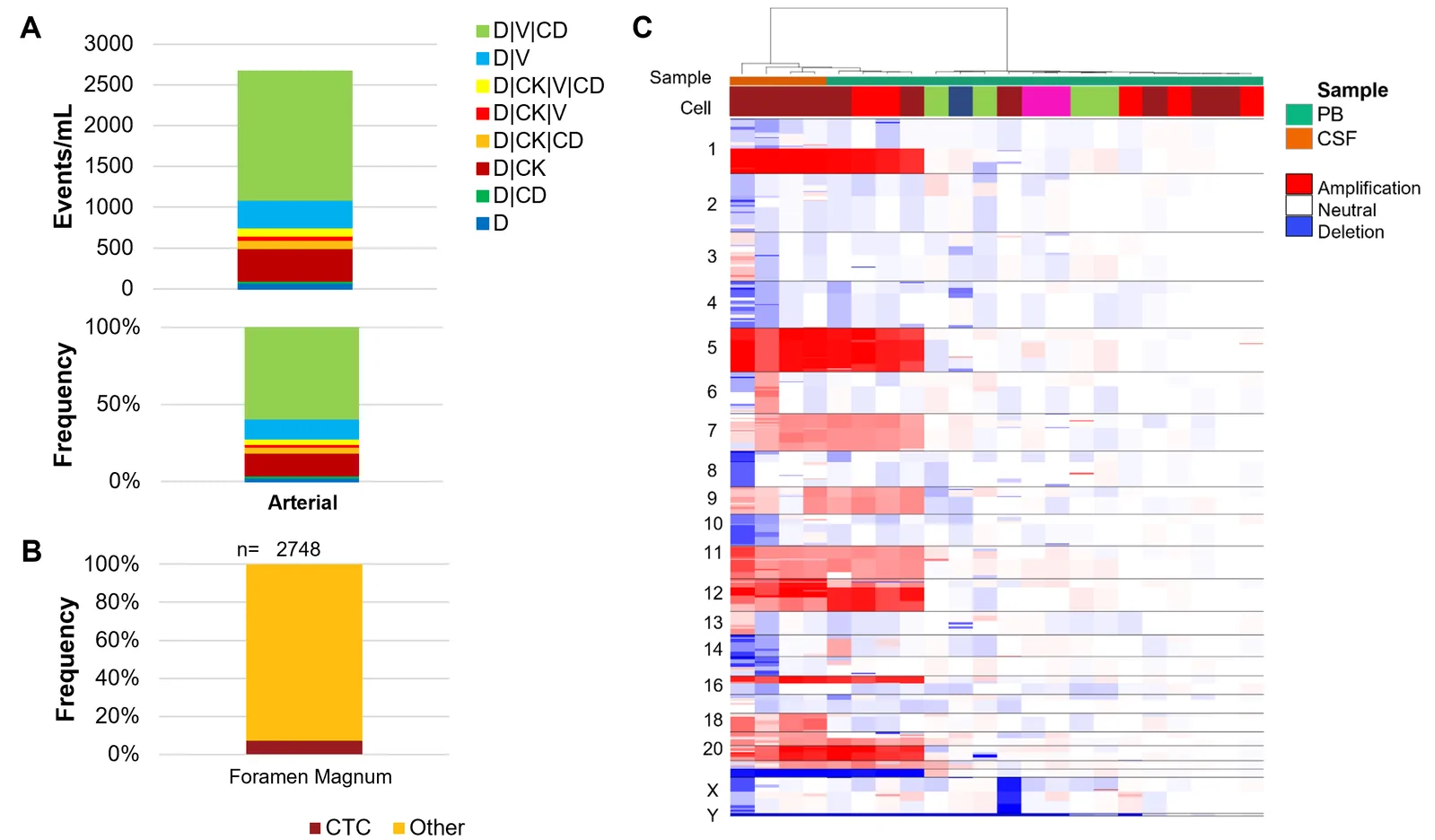
Patient 5 liquid biopsy analyses. (**A**) Counts (cell/mL) and frequency of rare cells found in the PB. (**B**) Frequency of all cells identified in the CSF. Cellular classifications are based on the expression profile of biomarkers (CK, VIM, CD) in the four fluorescent channels. (**C**) Heatmap and phylogenetic tree of single-cell CNAs across the entire population of cells isolated. Color scheme as in Figure 3.

**Table 1:** Sample quality control flags.

Sample	Comment
LMD-0002 venous PB	Low WBC count; no epi.CTCs detected. May reflect agonal circulatory depletion or post-mortem dilution. Interpret with caution; arterial PB preferred for comparison.
LMD-0004 foramen magnum CSF	Sample stored overnight at 4 °C prior to shipment. Low CK signal in candidate CTCs may reflect antigen degradation. No genomic analysis performed. Findings not used to draw biological conclusions.
LMD-0003 Ommaya/foramen magnum CSF	3 CSF cells isolated but all failed single-cell genomic amplification. This most likely reflects the age of the cells at amplification combined with the very low number of cells recovered, rather than a broader sampling issue. Reported descriptively without genomic characterization.
All PM samples	Post-mortem collections performed within the Legacy program’s 6–8 h post-death window per protocol.

**Table 2:** Phenotypic classification of circulating rare cells detected by fWSI.

Name	Biomarker Expression	Cellular Description
epi.CTC	CK+/VIM-/CD-	Epithelial circulating tumor cell. Primary carcinoma phenotype.
mes.CTC	CK+/VIM+/CD-	Mesenchymal CTC. Hybrid epithelial–mesenchymal phenotype; associated with plasticity and invasiveness.
im.CTC	CK+/VIM-/CD45+	Immune-mimicry CTC. Tumor cell co-expressing leukocyte antigen; may reflect immune evasion or cell fusion.
pc.CTC	CK+/VIM-/CD31+	Platelet-coated CTC. CTC with adherent platelets; associated with immune escape and metastatic fitness.
pc.mes.CTC	CK+/VIM+/CD31+	Platelet-coated mesenchymal CTC.
CEC	CK(variable)/VIM+/CD31+	Circulating endothelial cell. Genomically neutral; TME-associated.
cCAF	CK-/VIM+/CD-	Non-epithelial rare cell; may include CAF-like or stromal cells. Genomically neutral; TME-associated.

**Table 3:** Patient demographics and clinical information. AM = ante-mortem; PM = post-mortem; PB = peripheral blood; CSF = cerebrospinal fluid; RT = radiation therapy; VUS = variant of uncertain significance; IT = intrathecal; FM = foramen magnum; LV = lateral ventricle; Dx: Diagnosed.

Patient ID	1	2	3	4	5
Tumor characteristics					
Year Dx	2017	2021	2019	2017	2013
Histology	Invasive lobular carcinoma	Invasive ductal carcinoma	Infiltrating ductal carcinoma	Infiltrating ductal carcinoma	Invasive lobular carcinoma
ER	>95%	Negative	Positive	Negative	>95%
PR	20%	Negative	Positive	Negative	>95%
HER2/neu	Neg (0)	Neg (0)	Positive	Positive	Neg (FISH)
Metastatic sites	Bone, lung, liver, CNS, lymphatics, muscle/skin, peritoneum	Lymphatics, CNS	Bone/spine, brain, LMD	Brain, liver, lung, bone	Brain, bone, liver, peritoneum
Key genomic alterations	ERBB2 L755S (10.5%), D769Y (0.4%), L786V (0.4%); PIK3CA H1047R (11.8%); CCND1 amp (high); PTEN F257L (2.5%) (ctDNA, 2020; see Section 3.4)	PALB2, DKC1	CHEK2 c.1036C > T (VUS)	HER2 amp, MAP3K1, TP53, AAMDC amp, FADD amp, PAK1 amp	MAP3K1 30.4%, DNMT3A 6.9%, ESR1 overexp.
LMD diagnosis and CNS treatment					
LMD confirmed	MRI + positive CSF cytology (September 2021)	MRI + clinical course (2022)	Suspected; MRI + symptoms	MRI + clinical course (2024)	Autopsy (occult in vivo; normal prior brain MRI)
Ommaya reservoir	Yes—October 2021	No	Yes—November 2022	No	No
Intrathecal therapy	Topotecan (April–May)	None	Trastuzumab (Herceptin) via Ommaya	Trastuzumab via lumbar puncture (April–May 2024); insufficient CNS control	None
Prior systemic therapy	Multiple lines (ER+/HER2+ regimens)	Chemotherapy (TNBC)	Anti-HER2 regimens	Anti-HER2 regimens (multiple lines)	Hormonal therapy (ER+/PR+)
Radiation	Prior cranial RT	Prior cranial RT	Prior cranial RT	Prior cranial RT (necrotic lesions noted on imaging)	None documented
Clinical outcome					
Cause of death	LMD progression	LMD progression	Pulmonary infection (non-cancer related)	CNS disease progression	Liver failure (metastatic disease)
Samples analyzed					
PB collections	2 AM venous; 1 PM arterial	1 PM arterial; 1 PM venous ^†^	1 AM venous; 1 PM arterial; 1 PM venous	2 PM arterial	1 PM arterial
CSF collections	R ventricle, L ventricle, 4th ventricle, FM (all PM)	R ventricle, L ventricle (both PM)	2 AM (Ommaya + lumbar); 2 PM (Ommaya + FM)	FM only (PM) ^‡^	FM only (PM)
Single cells for genomics	19 total (10 PB, 9 CSF)	32 total (24 PB, 8 CSF)	17 PB; 3 CSF (amplification failed) ^§^	None (insufficient candidates)	22 total (18 PB, 4 CSF)

^†^ Venous PB for LMD-0002: low WBC count; no epi.CTCs detected—possible agonal depletion. Interpret with caution. ^‡^ LMD-0004 foramen magnum CSF stored overnight at 4 °C prior to shipment; low CK signal may reflect antigen degradation rather than true absence of CTCs. ^§^ LMD-0003 CSF amplification failure: most likely reflects the age of the sample at processing combined with the very low number of cells recovered (*n* = 3), rather than a broader sampling issue.

**Table 4:** Cross-patient summary of key findings. PB = peripheral blood; CK+ = cytokeratin-positive; CTC = circulating tumor cell; PM = post-mortem; CSF = cerebrospinal fluid; CNV = copy number variation; AM = ante-mortem; epi.CTC = epithelial circulating tumor cell; mes.CTC = mesenchymal circulating tumor cell; R = right; L = left; FM = foramen magnum.

Patient	PB Total Rare Cells and CK+ CTCs (Arterial vs. Venous, PM)	CSF CTC Pattern	Intrathecal Therapy and CSF Response	CSF CNV Concordance	Technical Caveats
Patient 1	PM arterial only, AM venous only at an earlier, different timepoint (no same-timepoint paired draw)	R ventricle 11.4% > L 6.4% > 4th 4.5% > FM 0.6% (single-patient, full 4-site gradient)	Topotecan via Ommaya (Apr–May); CTCs persisted in R ventricle despite therapy	CSF concordant (9/9 cells); 1/10 PB epi.CTC shared CSF-like profile	None flagged
Patient 2	Arterial higher for both total rare cells and CK+ CTCs (one of two patients with a same-timepoint paired PM arterial/venous draw)	L and R ventricles both high and similar	None	CSF concordant across epi.CTC and mes.CTC (8/8 cells); 2/24 PB cells shared CSF-like profile	Venous PB low WBC count
Patient 3	Arterial higher for total rare cells; venous higher for CK+ CTCs specifically (the other of two patients with a same-timepoint paired PM arterial/venous draw)	AM (2nd sample) 0%; separate PM CSF (Ommaya/FM) 0.05–0.1%	Trastuzumab via Ommaya; low/controlled CSF CTCs	No dominant clonal population in PB; CSF cells failed amplification (likely sample age and low cell number)	CSF amplification failure
Patient 4	PM arterial only, two arterial draws: 3.4 and 9.4 epi.CTC/mL (no paired venous)	FM 0.02% (low CK signal)	Trastuzumab via lumbar puncture; insufficient CNS control despite 2 months’ therapy	Not performed (no cells isolated)	CSF stored overnight at 4 °C, potential antigen degradation
Patient 5	PM arterial only, 398.2 epi.CTC/mL (no paired venous)	FM only, 7.18%	None	CSF concordant (4/4 cells); PB predominantly neutral (18 cells)	None flagged

## Data Availability

Data supporting the findings of this study are available from the corresponding author upon reasonable request, subject to patient privacy regulations. All data discussed in this manuscript are included in the main manuscript text or Supplementary Materials. The imaging data are available through the BLOODPAC Portal, Accession ID “BPPDS-0000001” (https://portal.bloodpac.org/data/BPPDS-0000001, accessed on 8 September 2026).

## Supplementary Materials

The following supporting information can be downloaded at: https://www.mdpi.com/article/10.3390/cancers18193124/s1, Figure S1. Schematic of sampled anatomical compartments. (A) CSF compartments sampled, from ventricular (proximal) sites (left and right lateral ventricles) through the 3rd ventricle and cerebral aqueduct to the 4th ventricle, and on to the foramen magnum (distal); the Ommaya reservoir accesses the right lateral ventricle only. (B) Peripheral blood compartments sampled: venous (ante-mortem and post-mortem) and arterial (post-mortem only), shown relative to the heart/lungs and to peripheral tissue/systemic capillary beds, where arterial blood is sampled before tissue exchange and venous blood after. Center: the blood-brain/blood-CSF barrier that tumor cells cross between the peripheral blood and CSF compartments shown in (A,B). Schematic; not to anatomical scale; Table S1. Per-sample CTC data. Single-cell genomic sequencing was performed only on a subset of cells drawn from the sample(s) with the highest CTC or CK+ cell counts within each patient (see Methods).

## Abbreviations

The following abbreviations are used in this manuscript:

AM	Ante-mortem
BSA	Bovine serum albumin
cCAF	Cancer-associated fibroblast-like cell
CD31	Cluster of differentiation 31 (endothelial cell marker)
CD45	Cluster of differentiation 45 (hematopoietic cell marker)
CEC	Circulating endothelial cell-like cell
cfDNA	Cell-free DNA
CK	Cytokeratin (epithelial cell marker)
CNA	Copy number alterations
CNS	Central nervous system
CNV	Copy number variation
CSF	Cerebrospinal fluid
CTC	Circulating tumor cell
ctDNA	Circulating tumor DNA
DAPI	4′,6-diamidino-2-phenylindole
Dx	Diagnosed
epi.CTC	Epithelial circulating tumor cell
ER	Estrogen receptor
FM	Foramen magnum
fWSI	Fluorescence whole-slide imaging
HER2	Human epidermal growth factor receptor 2
im.CTC	Immune-like circulating tumor cell
IRB	Institutional Review Board
IT	Intrathecal
LMD	Leptomeningeal disease
LV	Lateral ventricle
mes.CTC	Mesenchymal circulating tumor cell
MRI	Magnetic resonance imaging
PB	Peripheral blood
PBS	Phosphate-buffered saline
pc.CTC	Platelet-coated circulating tumor cell
pc.mes.CTC	Platelet-coated mesenchymal circulating tumor cell
PM	Post-mortem
PR	Progesterone receptor
RT	Radiation therapy
TME	Tumor microenvironment
VIM	Vimentin (mesenchymal cell marker)
WBC	White blood cell
WGA	Whole-genome amplification
