# Supplementary material for: The Landscape of Tumor Dissemination in Leptomeningeal Disease: Mapping CTC Plasticity and Regional Heterogeneity Across Blood and CSF Compartments
Source: Cancers (Basel). 2026 Sep 26;18(19):3124. doi: 10.3390/cancers18193124 (PMC13664032; doi:10.3390/cancers18193124)
Supplement: Supplementary file 1 [file cancers-18-03124-s001.zip › cancers-4532322-supplementary.pdf]

Supplementary Materials

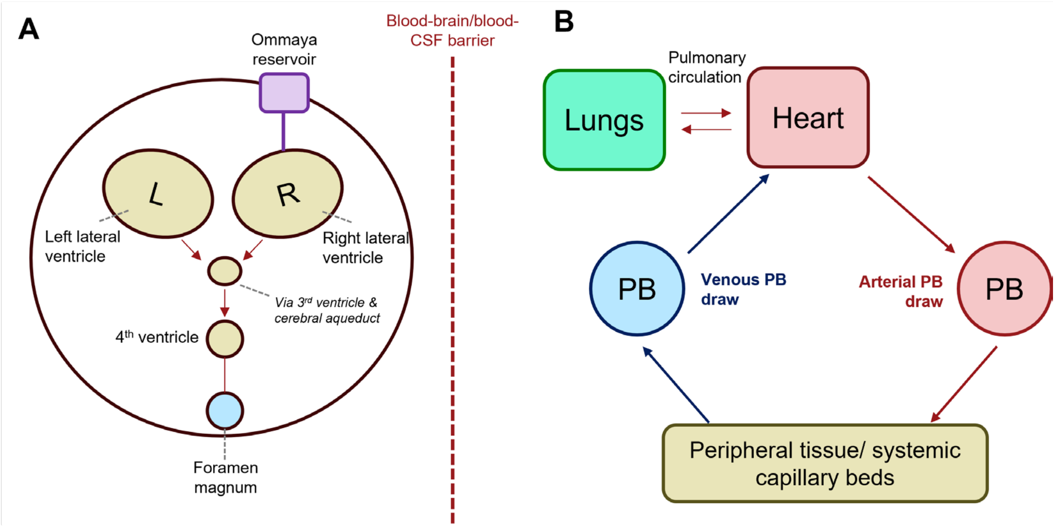

Supplementary Figure S1. Schematic of sampled anatomical compartments. (A) CSF compartments sampled, from ventricular (proximal) sites (left and right lateral ventricles) through the 3rd ventricle and cerebral aqueduct to the 4th ventricle, and on to the foramen magnum (distal); the Ommaya reservoir accesses the right lateral ventricle only. (B) Peripheral blood compartments sampled: venous (ante-mortem and post-mortem) and arterial (post-mortem only), shown relative to the heart/lungs and to peripheral tissue/systemic capillary beds, where arterial blood is sampled before tissue exchange and venous blood after. Center: the blood-brain/blood-CSF barrier that tumor cells cross between the peripheral blood and CSF compartments shown in (A) and (B). Schematic; not to anatomical scale.

Supplementary Table S1. Per-sample CTC data. *Single-cell genomic sequencing was performed only on a subset of cells drawn from the sample(s) with the highest CTC or CK+ cell counts within each patient (see Methods).*

Patient	Compartment	Timepoint	Sample type	CTC metric (as reported)
Patient 1	PB, venous	AM	Ante-mortem	19.16 epi.CTC/mL
Patient 1	PB, venous	AM	Ante-mortem	45.86 epi.CTC/mL
Patient 1	PB, arterial	PM	Post-mortem	27.68 epi.CTC/mL
Patient 1	CSF, R lateral ventricle	PM	Post-mortem	11.4%
Patient 1	CSF, L lateral ventricle	PM	Post-mortem	6.4%
Patient 1	CSF, 4th ventricle	PM	Post-mortem	4.5%
Patient 1	CSF, foramen magnum	PM	Post-mortem	0.6%
Patient 2	PB, arterial	PM	Post-mortem	1,425.73 epi.CTC/mL
Patient 2	PB, venous	PM	Post-mortem	0 epi.CTC/mL (low WBC, QC flag)
Patient 2	CSF, R lateral ventricle	PM	Post-mortem	34.0%; epi. + mes. CTCs
Patient 2	CSF, L lateral ventricle	PM	Post-mortem	38.3%; epi. + mes. CTCs
Patient 3	PB, venous	AM	Ante-mortem	2.98 epi.CTC/mL
Patient 3	PB, arterial	PM	Post-mortem	69.82 epi.CTC/mL
Patient 3	PB, venous	PM	Post-mortem	88.40 epi.CTC/mL
Patient 3	CSF, Ommaya + foramen magnum	PM	Post-mortem	0.05–0.1%
Patient 4	PB, arterial (draw 1)	PM	Post-mortem	3.4 epi.CTC/mL
Patient 4	PB, arterial (draw 2)	PM	Post-mortem	9.4 epi.CTC/mL
Patient 4	CSF, foramen magnum	PM	Post-mortem	0.02% (low CK, QC flag)
Patient 5	PB, arterial	PM	Post-mortem	398.2 epi.CTC/mL

Patient	Compartment	Timepoint	Sample type	CTC metric (as reported)
Patient 5	CSF, foramen magnum	PM	Post-mortem	7.18%
